# Supplementary material for: Hybrid Models and Biological Model Reduction with PyDSTool
Source: PLoS Comput Biol. 2012 Aug 9;8(8):e1002628. doi: 10.1371/journal.pcbi.1002628 (PMC3415397; doi:10.1371/journal.pcbi.1002628)
Supplement: Text S4 — Complete source code for the PyDSTool package (version 0.88.120504). Includes API documentation and help files linking to web pages. This file is identical to the current public release on Sourceforge.net. (ZIP) [file pcbi.1002628.s004.zip › PyDSTool/html/PyDSTool.Toolbox.optimizers.defaults-pysrc.html]

xml version="1.0" encoding="ascii"?


PyDSTool.Toolbox.optimizers.defaults


| Home | Trees | Indices | Help | PyDSTool |
| --- | --- | --- | --- | --- |

Package PyDSTool ::
Package Toolbox ::
Package optimizers ::
Module defaults

[hide private]

[frames] | no frames]

# Source Code for Module PyDSTool.Toolbox.optimizers.defaults

```
 1   
 2  # Matthieu Brucher 
 3  # Last Change : 2007-08-24 15:03 
 4   
 5  """ 
 6  Defines the defaults parameters for the generic optimizer framework 
 7  """ 
 8   
 9  __all__ = ['parameters', 'errors'] 
10   
11  SMALL_DF = 2 
12  SMALL_DELTA_X = 3 
13  SMALL_DELTA_F = 4 
14  SMALL_DELTA_X_X = 5 
15  SMALL_DELTA_F_F = 6 
16  FVAL_IS_ENOUGH = 10 
17  SOLVED_WITH_UNIMPLEMENTED_OR_UNKNOWN_REASON = 1000 
18   
19  IS_NAN_IN_X = -4 
20  IS_LINE_SEARCH_FAILED = -5 
21  IS_MAX_ITER_REACHED = -7 
22  IS_MAX_CPU_TIME_REACHED = -8 
23  IS_MAX_TIME_REACHED = -9 
24  IS_MAX_FUN_EVALS_REACHED = -10 
25  IS_ALL_VARS_FIXED = -11 
26   
27  FAILED_WITH_UNIMPLEMENTED_OR_UNKNOWN_REASON = -1000 
28   
29  parameters = { 
30                'alpha_step' : 1., 
31                'ftol' : 0.001, 
32                'gtol' : 0.001, 
33                'iterations_max' : 1000, 
34                'min_alpha_step' : 1., 
35                'xtol' : 0.001, 
36                } 
37   
38  errors = { 
39            SMALL_DF : "gradient norm is small enough", 
40            SMALL_DELTA_X : "absolute X difference is small enough", 
41            SMALL_DELTA_F : "absolute F(X) difference is small enough", 
42            SMALL_DELTA_X_X : "relative X difference is small enough", 
43            SMALL_DELTA_F_F : "relative F(X) difference is small enough", 
44            FVAL_IS_ENOUGH : "F(X) is small enough", 
45            SOLVED_WITH_UNIMPLEMENTED_OR_UNKNOWN_REASON : "Unknown reason of convergence", 
46   
47            FAILED_WITH_UNIMPLEMENTED_OR_UNKNOWN_REASON : "Unknown reason of failure", 
48            } 
49
```

  


| Home | Trees | Indices | Help | PyDSTool |
| --- | --- | --- | --- | --- |

|  |  |
| --- | --- |
| Generated by Epydoc 3.0.1 on Fri May 4 15:24:21 2012 | http://epydoc.sourceforge.net |
